# Supplementary material for: Pyruvate Dehydrogenase Kinase 1 inhibition mediated oxidative phosphorylation enhancement in cartilage promotes osteoarthritis progression
Source: BMC Musculoskelet Disord. 2023 Jul 20;24:597. doi: 10.1186/s12891-023-06585-6 (PMC10357736; doi:10.1186/s12891-023-06585-6)
Supplement: Supplementary file 1 — Additional file 1. [file 12891_2023_6585_MOESM1_ESM.zip › KEGG.Gsea.1653622432464/KEGG_MTOR_SIGNALING_PATHWAY.html]

Details for gene set KEGG\_MTOR\_SIGNALING\_PATHWAY[GSEA]

|  || Dataset | 55457\_collapsed\_to\_symbols.cls.cls #OA\_versus\_normal.cls.cls #OA\_versus\_normal\_repos |
| Phenotype | cls.cls#OA\_versus\_normal\_repos |
| Upregulated in class | normal |
| GeneSet | KEGG\_MTOR\_SIGNALING\_PATHWAY |
| Enrichment Score (ES) | -0.38717854 |
| Normalized Enrichment Score (NES) | -1.4644673 |
| Nominal p-value | 0.04180602 |
| FDR q-value | 0.20520273 |
| FWER p-Value | 0.967 |
Table: GSEA Results Summary

  

Fig 1: Enrichment plot: KEGG\_MTOR\_SIGNALING\_PATHWAY      
 Profile of the Running ES Score & Positions of GeneSet Members on the Rank Ordered List

  

| SYMBOL | TITLE | RANK IN GENE LIST | RANK METRIC SCORE | RUNNING ES | CORE ENRICHMENT || 1 | MAPK3 | mitogen-activated protein kinase 3 [Source:HGNC Symbol;Acc:HGNC:6877] | 757 | 0.579 | -0.0226 | No |
| 2 | AKT1 | AKT serine/threonine kinase 1 [Source:HGNC Symbol;Acc:HGNC:391] | 1192 | 0.478 | -0.0264 | No |
| 3 | EIF4E2 | eukaryotic translation initiation factor 4E family member 2 [Source:HGNC Symbol;Acc:HGNC:3293] | 1301 | 0.458 | -0.0061 | No |
| 4 | PIK3CB | "phosphatidylinositol-4,5-bisphosphate 3-kinase catalytic subunit beta [Source:HGNC Symbol;Acc:HGNC:8976]" | 2186 | 0.340 | -0.0536 | No |
| 5 | VEGFC | vascular endothelial growth factor C [Source:HGNC Symbol;Acc:HGNC:12682] | 2239 | 0.335 | -0.0367 | No |
| 6 | MLST8 | "MTOR associated protein, LST8 homolog [Source:HGNC Symbol;Acc:HGNC:24825]" | 2259 | 0.331 | -0.0174 | No |
| 7 | IGF1 | insulin like growth factor 1 [Source:HGNC Symbol;Acc:HGNC:5464] | 2286 | 0.328 | 0.0012 | No |
| 8 | ULK2 | unc-51 like autophagy activating kinase 2 [Source:HGNC Symbol;Acc:HGNC:13480] | 2796 | 0.270 | -0.0215 | No |
| 9 | STRADA | STE20 related adaptor alpha [Source:HGNC Symbol;Acc:HGNC:30172] | 3210 | 0.229 | -0.0393 | No |
| 10 | RPS6KA1 | ribosomal protein S6 kinase A1 [Source:HGNC Symbol;Acc:HGNC:10430] | 3389 | 0.213 | -0.0398 | No |
| 11 | INS | insulin [Source:HGNC Symbol;Acc:HGNC:6081] | 3885 | 0.166 | -0.0679 | No |
| 12 | CAB39 | calcium binding protein 39 [Source:HGNC Symbol;Acc:HGNC:20292] | 4181 | 0.140 | -0.0821 | No |
| 13 | CAB39L | calcium binding protein 39 like [Source:HGNC Symbol;Acc:HGNC:20290] | 5286 | 0.049 | -0.1649 | No |
| 14 | RPS6KB2 | ribosomal protein S6 kinase B2 [Source:HGNC Symbol;Acc:HGNC:10437] | 5496 | 0.033 | -0.1791 | No |
| 15 | EIF4EBP1 | eukaryotic translation initiation factor 4E binding protein 1 [Source:HGNC Symbol;Acc:HGNC:3288] | 5871 | 0.004 | -0.2080 | No |
| 16 | MAPK1 | mitogen-activated protein kinase 1 [Source:HGNC Symbol;Acc:HGNC:6871] | 6853 | -0.068 | -0.2801 | No |
| 17 | TSC2 | TSC complex subunit 2 [Source:HGNC Symbol;Acc:HGNC:12363] | 6946 | -0.075 | -0.2825 | No |
| 18 | RPS6KA3 | ribosomal protein S6 kinase A3 [Source:HGNC Symbol;Acc:HGNC:10432] | 7092 | -0.087 | -0.2883 | No |
| 19 | PIK3CD | "phosphatidylinositol-4,5-bisphosphate 3-kinase catalytic subunit delta [Source:HGNC Symbol;Acc:HGNC:8977]" | 7405 | -0.111 | -0.3056 | No |
| 20 | PIK3CA | "phosphatidylinositol-4,5-bisphosphate 3-kinase catalytic subunit alpha [Source:HGNC Symbol;Acc:HGNC:8975]" | 7733 | -0.136 | -0.3225 | No |
| 21 | PIK3CG | "phosphatidylinositol-4,5-bisphosphate 3-kinase catalytic subunit gamma [Source:HGNC Symbol;Acc:HGNC:8978]" | 7953 | -0.152 | -0.3301 | No |
| 22 | AKT3 | AKT serine/threonine kinase 3 [Source:HGNC Symbol;Acc:HGNC:393] | 8008 | -0.156 | -0.3245 | No |
| 23 | EIF4B | eukaryotic translation initiation factor 4B [Source:HGNC Symbol;Acc:HGNC:3285] | 8051 | -0.159 | -0.3178 | No |
| 24 | RPS6KA6 | ribosomal protein S6 kinase A6 [Source:HGNC Symbol;Acc:HGNC:10435] | 8071 | -0.161 | -0.3092 | No |
| 25 | BRAF | "B-Raf proto-oncogene, serine/threonine kinase [Source:HGNC Symbol;Acc:HGNC:1097]" | 8171 | -0.169 | -0.3063 | No |
| 26 | RPS6KA2 | ribosomal protein S6 kinase A2 [Source:HGNC Symbol;Acc:HGNC:10431] | 8397 | -0.185 | -0.3122 | No |
| 27 | VEGFB | vascular endothelial growth factor B [Source:HGNC Symbol;Acc:HGNC:12681] | 8673 | -0.206 | -0.3207 | No |
| 28 | PIK3R3 | phosphoinositide-3-kinase regulatory subunit 3 [Source:HGNC Symbol;Acc:HGNC:8981] | 8801 | -0.217 | -0.3170 | No |
| 29 | PIK3R5 | phosphoinositide-3-kinase regulatory subunit 5 [Source:HGNC Symbol;Acc:HGNC:30035] | 8804 | -0.217 | -0.3035 | No |
| 30 | PRKAA2 | protein kinase AMP-activated catalytic subunit alpha 2 [Source:HGNC Symbol;Acc:HGNC:9377] | 8864 | -0.222 | -0.2942 | No |
| 31 | EIF4E | eukaryotic translation initiation factor 4E [Source:HGNC Symbol;Acc:HGNC:3287] | 9709 | -0.293 | -0.3415 | No |
| 32 | ULK1 | unc-51 like autophagy activating kinase 1 [Source:HGNC Symbol;Acc:HGNC:12558] | 9798 | -0.301 | -0.3295 | No |
| 33 | RPS6KB1 | ribosomal protein S6 kinase B1 [Source:HGNC Symbol;Acc:HGNC:10436] | 10224 | -0.343 | -0.3410 | No |
| 34 | DDIT4 | DNA damage inducible transcript 4 [Source:HGNC Symbol;Acc:HGNC:24944] | 10818 | -0.407 | -0.3617 | Yes |
| 35 | PIK3R2 | phosphoinositide-3-kinase regulatory subunit 2 [Source:HGNC Symbol;Acc:HGNC:8980] | 10956 | -0.425 | -0.3457 | Yes |
| 36 | VEGFD | vascular endothelial growth factor D [Source:HGNC Symbol;Acc:HGNC:3708] | 11095 | -0.443 | -0.3287 | Yes |
| 37 | PIK3R1 | phosphoinositide-3-kinase regulatory subunit 1 [Source:HGNC Symbol;Acc:HGNC:8979] | 11099 | -0.443 | -0.3011 | Yes |
| 38 | STK11 | serine/threonine kinase 11 [Source:HGNC Symbol;Acc:HGNC:11389] | 11451 | -0.496 | -0.2973 | Yes |
| 39 | PRKAA1 | protein kinase AMP-activated catalytic subunit alpha 1 [Source:HGNC Symbol;Acc:HGNC:9376] | 11499 | -0.501 | -0.2696 | Yes |
| 40 | TSC1 | TSC complex subunit 1 [Source:HGNC Symbol;Acc:HGNC:12362] | 11575 | -0.511 | -0.2434 | Yes |
| 41 | RPS6 | ribosomal protein S6 [Source:HGNC Symbol;Acc:HGNC:10429] | 11662 | -0.526 | -0.2171 | Yes |
| 42 | HIF1A | hypoxia inducible factor 1 subunit alpha [Source:HGNC Symbol;Acc:HGNC:4910] | 11833 | -0.556 | -0.1955 | Yes |
| 43 | PDPK1 | 3-phosphoinositide dependent protein kinase 1 [Source:HGNC Symbol;Acc:HGNC:8816] | 11839 | -0.557 | -0.1610 | Yes |
| 44 | MTOR | mechanistic target of rapamycin kinase [Source:HGNC Symbol;Acc:HGNC:3942] | 12047 | -0.605 | -0.1391 | Yes |
| 45 | VEGFA | vascular endothelial growth factor A [Source:HGNC Symbol;Acc:HGNC:12680] | 12629 | -0.813 | -0.1334 | Yes |
| 46 | PGF | placental growth factor [Source:HGNC Symbol;Acc:HGNC:8893] | 12850 | -1.059 | -0.0842 | Yes |
| 47 | AKT2 | AKT serine/threonine kinase 2 [Source:HGNC Symbol;Acc:HGNC:392] | 12896 | -1.404 | 0.0003 | Yes |
Table: GSEA details [plain text format]

  

Fig 2: KEGG\_MTOR\_SIGNALING\_PATHWAY      
 Blue-Pink O' Gram in the Space of the Analyzed GeneSet

  

Fig 3: KEGG\_MTOR\_SIGNALING\_PATHWAY: Random ES distribution      
 Gene set null distribution of ES for **KEGG\_MTOR\_SIGNALING\_PATHWAY**
